# Autism and the right to education in the EU: Policy mapping and scoping review of the United Kingdom, France, Poland and Spain

**DOI:** 10.1371/journal.pone.0202336

**Published:** 2018-08-30

**Authors:** Monika Roleska, Andres Roman-Urrestarazu, Sarah Griffiths, Amber N. V. Ruigrok, Rosemary Holt, Robin van Kessel, Kathleen McColl, William Sherlaw, Carol Brayne, Kasia Czabanowska

**Affiliations:** 1 Department of International Health, School CAPHRI, Faculty of Health, Medicine and Life Sciences, Maastricht University, Maastricht, the Netherlands; 2 Institute of Public Health, University of Cambridge, Cambridge, United Kingdom; 3 Autism Research Centre, Department of Psychiatry, University of Cambridge, Cambridge, United Kingdom; 4 Département des sciences humaines, sociales et des comportements de santé, Ecole des hautes études en santé publique, Rennes, France; 5 Institute of Public Health, Faculty of Health Sciences, Jagiellonian University, Krakow, Poland; University of Illinois at Urbana-Champaign, UNITED STATES

## Abstract

**Introduction:**

Autistic people may have different educational needs that need to be met to allow them to develop their full potential. Education and disability policies remain within the competence of EU Member States, with current educational standards and provisions for autistic people implemented locally. This scoping review aims to map EU and national special education policies with the goal of scoping the level of fulfilment of the right to education of autistic people.

**Methods:**

Four EU countries (United Kingdom, France, Poland and Spain) were included in this scoping review study. Governmental policies in the field of education, special education needs and disability law were included. Path dependency framework was used for data analysis; a net of inter-dependencies between international, EU and national policies was created.

**Results and discussion:**

Each country created policies where the right to free education without discrimination is provided. Poland does not have an autism specific strategy, whereas the United Kingdom, France and Spain have policies specifically designed for autistic individuals. Within the United Kingdom, all countries created different autism plans, nevertheless all aim to reach the same goal—inclusive education for autistic children that leads to the development of their full potential.

**Conclusion:**

Policy-making across Europe in the field of education has been changing through the years in favour of autistic people. Today their rights are noticed and considered, but there is still room for improvement. Results showed that approaches and policies vastly differ between countries, more Member States should be analysed in a similar manner to gain a broader and clearer view with a special focus on disability rights in Central and Eastern Europe.

## 1. Introduction

Autism Spectrum Conditions (ASC) are characterized by difficulties with social interaction, verbal and non-verbal communication, and restricted and repetitive behaviour; which are usually present since early childhood and continue during the life course [1]. The prevalence of autism has been steadily increasing, which in part, likely reflects changes in the diagnostic concept and criteria as well as greater awareness, better recognition, changes in the use of diagnosis, and earlier age of diagnosis [2–4]. The current median prevalence of autism across the globe is 0.62–0.70% [5], although the latest large-scale surveys report estimates of 1–2%. Similar rate is found in adults and with approximately 40% of autism cases being undiagnosed [2,6]. One of the main characteristics of this condition is the diversity with which symptoms appear and develop in those affected [1].

ASC has functional and financial impact on those affected and their families [7–9]. This impact ranges from high health expenditure and out of pocket payments for non-covered health services to low employment prospects, poor mental health, anxiety and wellbeing problems [7–9]. In order to tackle these problems and increase quality of life, autonomy, and insertion in society, the exercise of the fundamental right to education for autistic people in the European Union (EU) is crucial[10,11]. Due to the diversity of presentation and evolution of ASC, the educational needs of autistic people tend to differ across time and between individuals [11]. Provision for Special Education Needs (SEN) should be provided from early childhood, throughout the school years and extend to lifelong education to ensure that the full potential of people living with ASC in the EU is reached [1,12]. Regardless of the severity of the condition, inclusive education of autistic people has been endorsed both through the Universal Declaration of Human Rights (UDHR) (Article 26) [13] and the Convention on the Rights of Persons with Disabilities (CRPD) [14], which has been signed by all EU countries. The CRPD is the first international, legally-binding Human Rights treaty that focuses solely on people with disabilities [14]. Member States that sign this treaty have the obligation to address problems and respect the needs of people with disabilities, including autism, such as the right to education. Specifically, the treaty sets out the right for all people with disabilities to be included in the general education system and receive required support (Article 24)[14]. Furthermore, Article 31 and 33 of the Convention lay down general conditions for the monitoring of the Convention specific obligations on States parties to monitor how the rights laid down in the Convention are being respected.

Although several areas of law are harmonized in the EU, public health remains within the competence of the Member States (Article 168)[15]. In this paper we hypothesize that the lack of harmonization in public health and education policy and common standard of policy making in EU countries has resulted in a gap of delivery of SEN provision and the fulfilment of the right to education of autistic people across Europe. In order to evaluate this we included four European countries, namely the United Kingdom (UK), France, Poland and Spain, countries as part of a wider project to map all 28 EU Member States SEN and ASC policy; aiming to create a comparative policy analysis that will culminate in a report of the differences that might be observed in ASC policy across the EU. Additionally, these countries were chosen based on the EDUCAUS which is a European consortium of autism policy researchers from the countries under study that took part in this research. The work carried out in this scoping review aims to first map International, EU and national ASC policies in the field of education across these four countries, to then expand all 28 EU Member States as part of a larger project. We use a policy path dependency methodology that links both the precedents of the UDHR and CRPD as main theoretical frameworks of reference. We aim to show main policy outcomes and the levels of interdependency between the different layers of policy developments to gain a clear view on the level of fulfilment of right to education and public health impact of autistic people across four EU countries.

## 2. Methodology

We chose to apply educational policy path dependency analysis using qualitative methods in the form of a scoping review to explore and analyse the links between education and human rights. We used a path dependence approach to our work in order to explain how at both the EU and local level the set of decisions that policy makers have faced in the field of ASC policy has been limited by the previous decisions and policy frameworks enacted in the past. This analysis will be based on a set of critical junctures and reinforcing reactive sequences as proposed by Ruth and David Collier in their work [16–18]. This methodology is suited for exploring research questions about policy pathways that are based on precedent legislation that pose critical junctures in ASC policy such as the UDHR [19]. It also allows us to conceptualize path dependence in ASC policy as a social process grounded in a dynamic of “increasing returns” [17]. Furthermore, it integrates competing ideas and values allowing exploration of the interactions among different countries and how they follow supranational guidance, such as guidance from the EU. Our purposive sample was chosen to facilitate a constant-comparative approach to policy and legislative data analysis, derived from path dependency. In the absence of a single, nationally representative data source in the EU, we adopted a modular approach to legislative and policy work across the different educational policy layers of analysis (International, EU and National).

### 2.1 Theoretical framework for path dependency

The use of a path dependence methodology enables for policy processes to be traced. It aims to (1) clarify what factors play a role in critical policy junctures, (2) create a reference framework and describe how decision-making processes come to conclusions, and (3) explain how stakeholder behaviour that occurs as a response to changes in the external environment is affected [17,20]. In this study, the UDHR was the critical juncture policy (1948), a breakthrough document that greatly swayed the creation of the EU and the content of its policies and that of its Member States. Time and policy were two variables depicted on a timeline to show their interdependency to enable further analysis. This allowed us to view policy creation as a historical sequence and identify path dependence [19]. Current disability and autism policies are a result of precedent events that were tracked with this framework. All included policies were analysed by determining their input in the field of education, advantages and disadvantages, as well as their effect on other policies.

### 2.2 Data collection and search strategy

We included the UK (64.72 million people) (divided between England, Wales, Scotland and Northern Ireland), France (64.40 million people), Poland (38.61 million people) and Spain (46.12 million people) [21] as a sample of different policy initiatives across the EU and also considering that these countries represents a large percentage (42.36%) of the total EU population and this would allow us to comparatively asses a large proportion of people that might be affected in the EU by ASC and SEN policy. Moreover, the choice of countries allowed for an analysis across the EU, since it includes one Western EU country used as reference point (the UK) and one Western EU country used as comparison (France), one Eastern EU (Poland), and one Southern EU country (Spain) [22]. Among these countries, Spain and the UK operate under different levels of devolution and legislative systems, where the central government and the sub-regions and constituent countries each have their respective devolved policy areas they are responsible for [23,24]. We chose these countries to compare how devolution has affected autism policy. On the other hand, Poland and France operate a centralised legislative system, where the central government legislates for the entire nation [25,26], which is the reason why we chose them as case studies. We included these four countries as the initial step to map all 28 EU Member States ASC policy, work that will be carried out in parallel and will result in an EU wide report. The scope of ASC policies was narrowed to only include policies that relate to the national education system, the right to education, special needs education, and disability laws. Additionally, policies and documents relating to autism and educational policy of those younger than 18 years, with any comorbid health condition in any setting were eligible for inclusion. Legal documents provided by governments were included, whereas programs and strategies developed by non-governmental organizations were excluded. Furthermore, legislation was eligible for inclusion as long as it was published after 1945. Constitutions were included regardless of publication date, because of their fundamental role in legislation.

The first step or identification stage was to extract relevant data from governmental policies and legislation that address the right to education of autistic people. Data was taken directly from their original sources in legal documents from the four countries included in this scoping review. No limits were put on language and foreign documents that were not translated into English. There was just one governmental source for the United Nations (http://www.ohchr.org), two for the EU (http://eur-lex.europa.eu; http://www.europarl.europa.eu), five for the UK and its constituent countries (http://www.legislation.gov.uk; http://www.gov.scot; http://gov.wales; http://www.wales.nhs.uk; https://www.health-ni.gov.uk), three for Poland (http://dziennikustaw.gov.pl; http://orka.sejm.gov.pl; http://www.sejm.gov.pl), two for France (http://www.conseil-constitutionnel.fr; http://www.cnsa.fr) and two for Spain (http://www.congreso.es;https://www.msssi.gob.es/). Moreover, no time limit was used during the searches, as the goal was to create a timeline of policies. The second step was to develop a multilevel search strategy that integrates policy and academic publications to search through electronic databases (PubMed, Google Scholar) and the abovementioned governmental sources using a combination of text words and index terms. Words included in our search were “autism”; “disability”; “SEN”; “education”; “law”; “policy” and the following combinations “autism & law”; “autism & policy”; “autism & SEN”; “autism & education”; “autism & disability”; “SEN & policy”; “SEN & law”; “disability & law”; “disability & policy”. We adapted the search strategy for each database, for example through multiple translations of the index terms so that lingual differences were accounted for as much as possible. Then, in a third step of screening, we amalgamated both policy and academic publications. Legal and policy documents provided by the governments were included after testing in the eligibility stage, whereas programs and strategies developed by non-governmental organizations were excluded. The fourth step of our search strategy was to obtain further information through searching reference lists and grey literature. Policy documents and governmental strategies in selected countries were now compared to and aligned with the EU disability and educational policy. In case mentioned documents were not present, general disability policies and legislation were analysed. This was built on an independent appraisal of three searches: one looking for ASC and educational policy in the international level, one for the EU and one looking for the individual countries. The fifth step was merging these three geographical searches into a single data repository for the purpose of our scoping review. All data collection and analysis were based at the Autism Research Centre and Institute of Public Health University of Cambridge.

### 2.3 Data analysis and path dependency

An analysis of policy path inter-dependencies between current and past EU and national policies in the field of education and ASC was performed. Path dependence technique enables to identify patterns of policy-making and establish influences and inter-relations between policies in linear layers of temporal policy [17,19]. It also allows for policy process-tracing, aiming at elucidating what factors are present in critical policy junctures for (a) creating a reference framework, (b) how this decision process comes to conclusions, and (c) the actual behaviour that then occurs across different stakeholders in reaction to this and (d) how it affects institutional arrangements [17,18]. In this case, initial policy was the UDHR (1948), a milestone document that influenced both the creation and the content of EU as well as national policies that then went in the direction of recognizing and considering fundamental human rights. Time and policy were two variables presented on a timeline to show their linkage and overlap to facilitate further analysis. This enabled us to see policy creation as historical sequences and patterns and identify path dependence [19]. Current disability and autism policies are a result of previous events that were tracked using this framework. Each policy was analysed by identifying its input in the field of education, pros and cons, as well as relation to other policies.

## 3. Results

**Fig 1:**
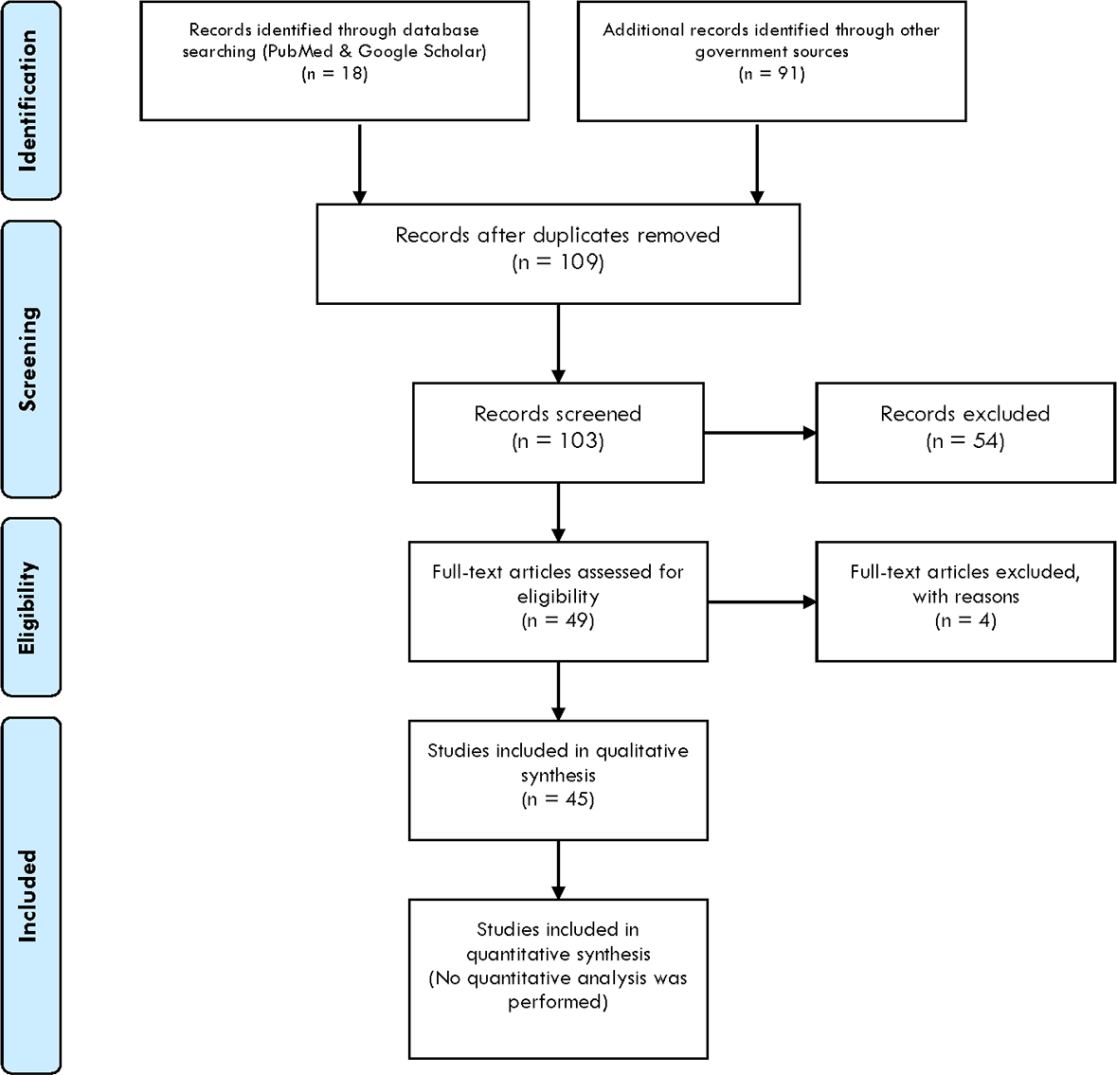
An overview of the search strategy depicted in PRISMA flowchart.

**Fig 2:**
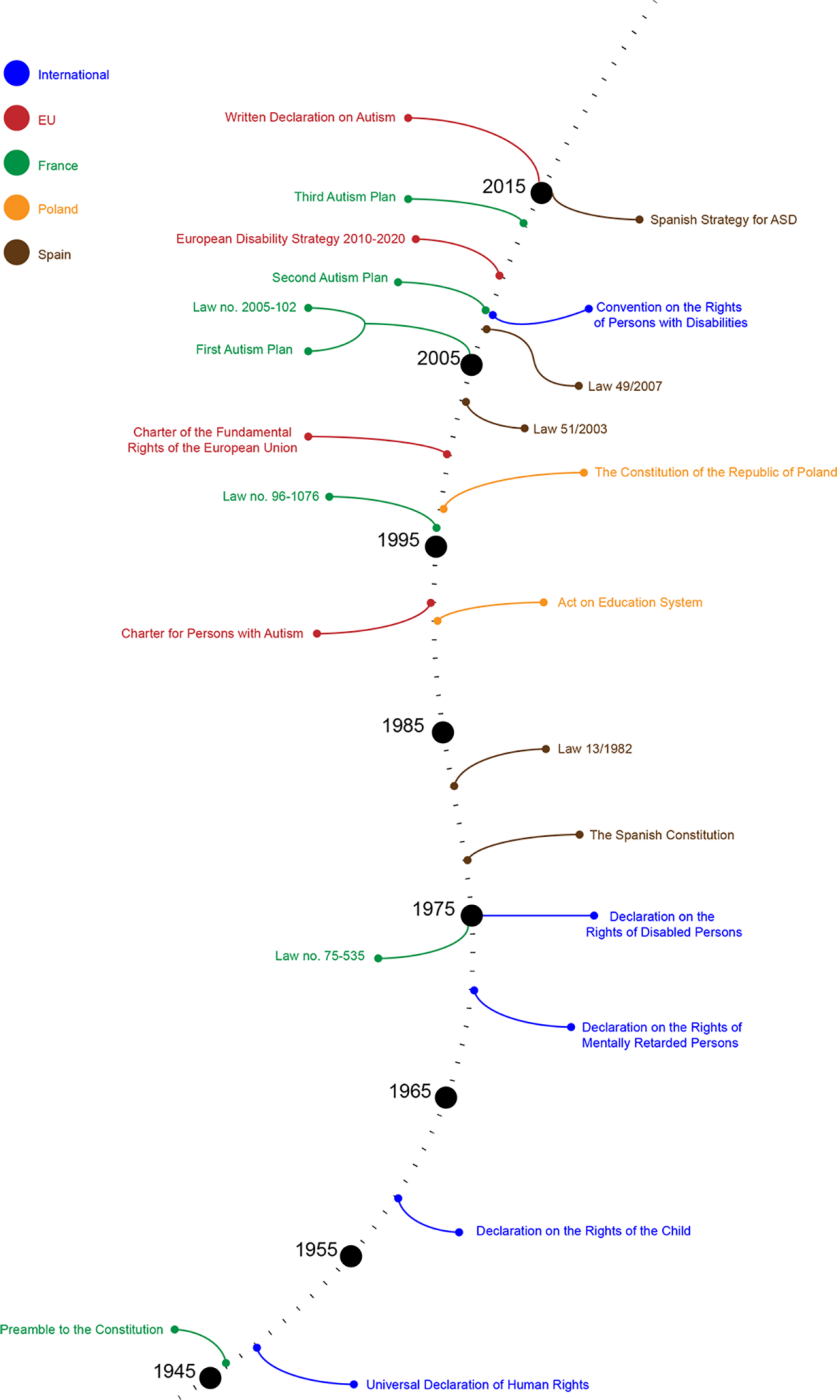
An overview of international, EU, French, Polish, and Spanish policies on SEN and autism.

**Fig 3:**
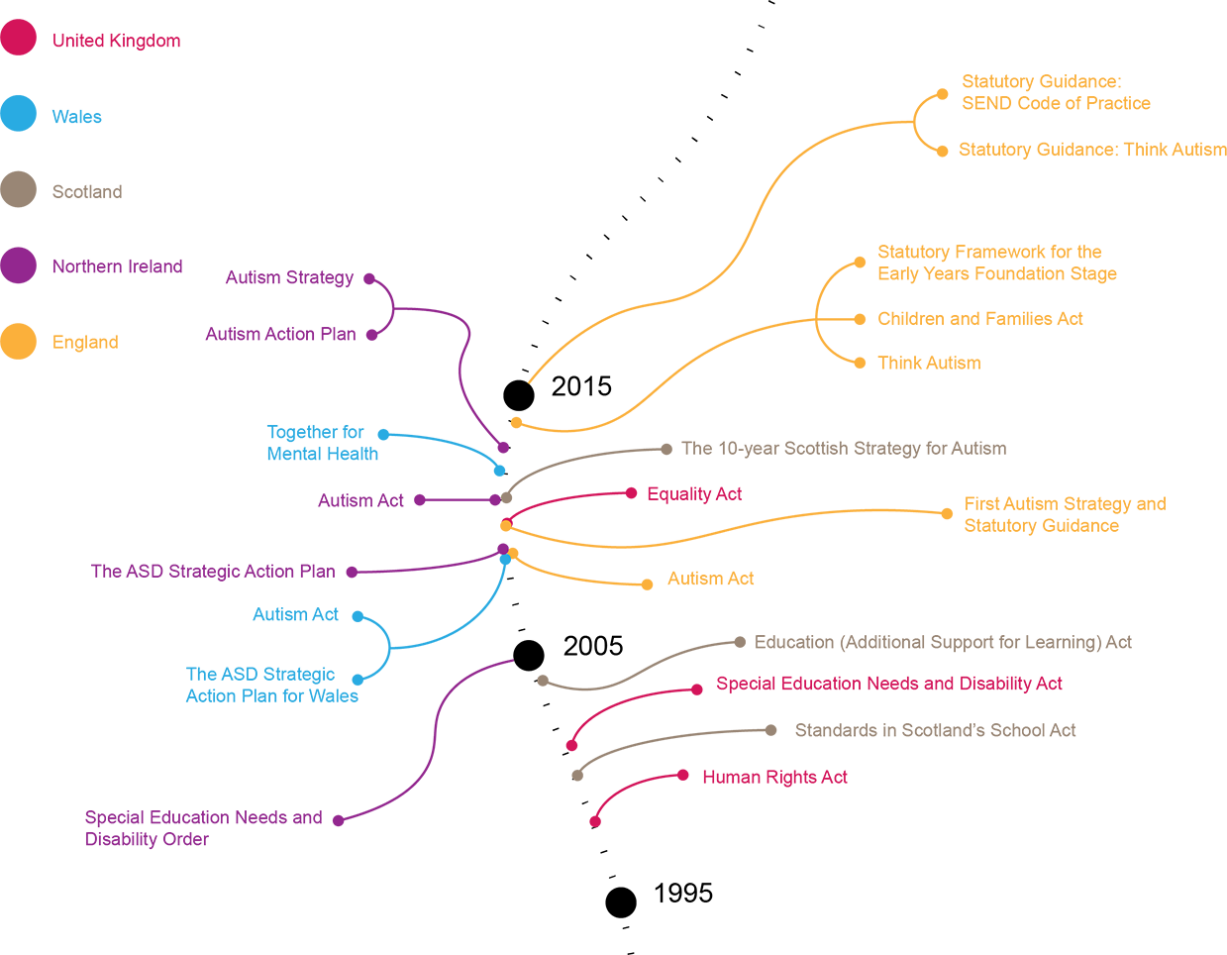
An overview of the SEN and autism policies in the UK and its constituent countries.

We identified 18 sources through database searches (PubMed and Google Scholar) and 91 additional records through governmental sources at the study identification stage. At this stage we observed no duplicates and therefore we included 109 studies and policy documents for the screening stage. During the screening of 103 records (6 records were excluded since they were different in scope), we excluded 54 records that did not meet our inclusion criteria after which we were able to include 49 records assessed for eligibility with four articles and/or policy documents excluded at this stage. The final number of sources included in our qualitative analysis was 45, all being policy documents. A full list of the policy documents used in this paper is available (S1 File). Please see our PRISMA flow chart (Fig 1) for the details of the results of the search strategy of this scoping review. With these inputs we created a timeline of policies for each country to present the results. Fig 2 shows policies relevant in the field of education internationally, in the EU, France, Poland and Spain, whereas Fig 3 covers the UK.

### 3.1 International level

The paramount act that mentions the right to education in modern times is UDHR [13]. In Article 26 it is stated that everyone has the right to education and that the aim of it should be to fully develop people’s potential [13]. Although the UDHR states that everyone, without distinctions, has fundamental rights they are entitled to, United Nations proclaimed that children require special care and assistance [27]. Therefore, shortly afterwards, in 1959, Declaration of the Rights of the Child was adopted by the United Nations. Principle 5 of the Declaration of the Rights of the Child states that if a child has special needs because of their mental or physical condition, appropriate treatment, education and care should be provided [27].

This declaration, along with the UDHR, require signatory countries to ensure all children have access to education that is provided without discrimination in relation to, for example, their disability or ASC diagnosis. After these two ground breaking Declarations, the Declaration on the Rights of Mentally Retarded Persons from 1971 was instituted which restates in Paragraph 1 that all people should have equal rights, and in Paragraph 2 that the right to education should be respected with the aim to fully develop potential for those with disabilities [28]. As the 1971 declaration only applied to persons with intellectual disabilities, a need to expand and define the scope of rights meant that in 1975 the Declaration on the Rights of Disabled Persons was signed, that in turn expanded the concept of disability and then proceeded to include all people with disabilities also clearly defining the term ‘disability’ [29]. The term *disabled person* was defined as ‘any person unable to ensure by himself or herself, wholly or partly, the necessities of a normal individual and/or social life, as a result of deficiency, either congenital or not, in his or her physical or mental capabilities’. Autistic people, therefore, are within this definition. All documents described above only lightly touch upon the right to education and do not state the type of education that should be provided and failed to define implementation strategies [13,27–29]. Nevertheless, because of these documents, countries were obliged to include and take into consideration the needs of persons with disabilities, including autism, when creating their legislation [13,27–29]. These international documents influenced most EU policies.

Article 24 of the CRPD that was signed by the United Nations in 2006 clearly explains and delineates in detail the type of education to be provided as well as the aims and support that should be given to people with disabilities [14]. The CRPD states that education should be provided without discrimination, be inclusive and aim at development of full potential, talents, personality and creativity [14]. Persons with disabilities cannot be excluded from mainstream schools and free, inclusive primary and secondary education should be granted [14]. There should also be an equal access to vocational training and adult education [14]. Although CRPD is an important document for autistic people, it does not directly mention autism, or other specific disabilities. This is one of the shortcomings of CRPD, as it does not give a definition of disability, but states it is an ‘evolving concept’ that can be treated very broadly, and be both an advantage and disadvantage. As the document sets rights for people with disabilities, a firm definition of what is meant by disability ought to be provided.

### 3.2 EU level

With this international discussion based on human rights, in 1992, the Charter for Persons with Autism was created by Autism-Europe [30]. This Charter directly referred to the Declarations, of Human Rights and on the Rights of Persons with Disabilities, in Point 3 of the Charter says autistic people should have access to appropriate education and Point 6 states the right to support that enables them to live to their fullest potential [30]. This document is very specific and directly mentions autistic people, therefore differs from previous documents that defined disability very broadly and made it difficult to clearly define the scope of their application in the sphere of education.

As the rights and freedoms that are protected in EU regulations were established through time in different forms, in 2000, they were collected into one, legally binding document–the Charter of Fundamental Rights of the European Union. Highlighting these rights by creating the charter aimed to enhance their protection and help create a closer union based on common values (Preamble) [31]. Article 14 states the right to education and vocational training for everyone, although autism is not explicitly mentioned [31].

In 2010, European Commission adopted the European Disability Strategy 2010–2020 that highlighted that inclusive education of children with disabilities is important and beneficial for their development [32]. Autistic People are not specifically mentioned by the strategy, and similar to the CRPD, disability is treated as one unified concept. As a result, the strategy aims to tackle the common needs and difficulties of persons with all forms of disabilities and conditions. This effort is put on inclusive education, which is consistent with CRPD and can be beneficial for autistic people.

In 2015, the Written Declaration on Autism was adopted by the European Parliament and co-signed by 418 Members of European Parliament. Point 3 of this Declaration states the importance of early diagnosis of autism to be able to provide appropriate support and education [33]. Moreover, in Point 5, Commission and Council were called upon to create an European Autism Strategy to ensure needs of people with the condition are met and to expand autism research and prevalence studies [33]. So far, no European Strategy for Autism has been developed, nevertheless, a conference was hosted by the European Parliament to establish what is needed to adopt such strategy.

### 3.3 United Kingdom

#### 3.3.1 England

Until now, the UK has implemented numerous policies that are relevant for autistic people in the field of education. However, many of these policies are not applicable in all of the UK because of different levels of devolution to the constituent countries. Out of the numerous pieces of UK legislation, Human Rights Act 1998 is an example that specifically mentions the right to education that shall be guaranteed to every human being (Part II, Article 2) [34]. On one hand that could give the UK Government a wide discretion to implement strategies in the most convenient or effective way. On the other end, lack of more structured approach to serving the interests of people with different disabilities implies less robust frameworks, with much less accountability on the side of the Government. In 2001, the Special Educational Needs and Disability Act was enacted, which states children with disabilities are able to attend mainstream schools (Part 1, 1) [35]. Moreover, people with disabilities cannot be discriminated against, special needs shall be respected and equal opportunities provided (Part 2, Chapter 1) [35].

The most important legislation for autistic people in the England and Wales is the Autism Act 2009, which, regardless of being an important step in the area of recognizing the rights of autistic people, applied only to adults. This was a major drawback, as ASC starts in early childhood and the need to ensure adequate education and support for children is crucial to secure insertion in society. The Parliament did not specify the reason as to why the Act did not include children, so it can be speculated that autistic children were at the time under protection of other documents, such as the Special Educational Needs and Disability Act. Due to the limited scope of the Autism Act, the strategies produced by the English Government [36,37], strategy mainly focused on improving autism diagnosis, helping adults with autism get employed and access support [36].

In 2010, Equality Act was passed by the UK Parliament (not applicable in Northern Ireland), that stipulated that children with disabilities should not be discriminated against (Article 88—Schedule 10) [38]. In 2014, Statutory Framework for the Early Years Foundation Stage came into force that was neither disability, not autism specific, nevertheless mentioned children with special needs and their rights in the field of education. According to this framework, special needs of children should be recognized and acted upon with specialist support (Paragraph 1.6) [39]. This Framework mentions that children have different abilities, therefore they have different needs and learning plans should to be targeted to areas where the child may perform weaker (Paragraphs 2.4, 2.10) [39]. This is important as it increases the chances of autistic children to complete the program and graduate, if education plans are tailored to their unique needs.

Shortly after this framework, Children and Families Act 2014 came into force in England. This Act is of high importance for autistic people as it introduced a Special Educational Needs and Disability (SEND) Support System that covers education, health and social care [40]. The Children and Families Act led the English Government to create another autism strategy, as the first one applied exclusively to adults. In 2014 England came up with their second autism strategy, called Think Autism. It highlighted the importance of education settings, such as the correct lighting as well as quiet areas as beneficial for autistic people (Paragraph 4.7) [41]. This Strategy also recommended increased training for staff that work with autistic people to broaden their knowledge and understanding to be able to adjust their behaviour when dealing with people with the condition (Paragraph 4.8) [41]. Processes of transition between different levels of education can be stressful for those with ASC, therefore the strategy proposed good transition planning in schools to address the needs of autistic people (Paragraph 11.2) [41]. The Strategy also refers to the Education, Health, and Care plans introduced by the Child and Families Act 2014, as these plans aim to be person-centred and bring benefits for autistic people (Paragraph 11.5) [41]. Moreover, The Department for Business Innovation and Skills (BIS) provides Learning Support funding to colleges to ensure any additional needs of autistic people are met so that they can participate fully in education (Paragraph 11.6) [41].

Closely related to the Think Autism was the Statutory Guidance that came a year after the Strategy. The Guidance additionally mentions that post-16 providers should offer courses that are challenging and help autistic students to improve (Paragraph 3.20) [42]. Traineeships are also mentioned to give autistic people skills needed for future work (Paragraph 3.24) [42].

Lastly, Statutory Guidance–Special Educational Needs and Disability Code of Practice: 0 to 25 years from 2015 introduced important rights for autistic people. Here, the right to education on equal basis with others was also mentioned (Paragraphs 1.26, 1.27, 1.28, and 4.37) [43]. Additionally, the aim should be to develop full potential and develop creativity, talents and ensure participation in society is enhanced (Paragraphs 1.22, 1.31, 1.34, 1.39, 6.1, 6.2, 7.6, and 7.13) [43].

Policies in England are well aligned with Article 24 of CRPD, equal access to inclusive education as well as the aims of education to develop potential and lead to independence are present in the documents described above. The importance of developing unique skills and creativity is also included. Individualised support for autistic people mentioned in CRPD is also in place in England.

#### 3.3.2 Wales

The Autism Act 2009 applied to England and Wales and governments of these countries came up with two separate strategies. The reason for that was arguably to comply the strategies with the specific demands of local environments that required more adjusted implementation plans. For the purposes of the work that was carried in this scoping review study, the common legal basis for both strategies allow for an adequate comparison of the results in both countries. The Autistic Spectrum Disorder (ASD) Action Plan for Wales was introduced in 2009 that set actions to be achieved within 10 years. This Strategy refers to previous policies and does not introduce any new rights, nevertheless is an important document for people with the condition as it is a summary of the rights they can exercise in Wales. The importance of appropriate education and its long-lasting positive effects were highlighted. Needs of autistic people vary, nevertheless they all have the same rights to education (Paragraph 5.2) [44]. School curriculum should be adapted and tailored to the person to provide successful education (Paragraph 5.3)[44]. Additional funding is given by the Welsh Government to ensure colleges are able to provide support for autistic people (Paragraph 5.10) [44]. There are traineeships and apprenticeships available for people with the condition to improve their chances of proceeding into employment (Paragraph 5.15) [44].

Welsh Government also came up with a 10-year strategy called Together Mental Health that does not specifically mention autism, nevertheless touches upon needs of persons with mental health problems. The strategy mentions the importance of increasing education and employment by cross-government approach (page 11) [45]. Moreover, education providers should ensure that early school experience is good and that support is provided (Technical Annex 3)[45].

#### 3.3.3 Scotland

Apart from legislation that is passed by the UK Parliament, there were several relevant policies that came into force in Scotland. Starting in 2000, Standards in Scotland’s Schools Act stated that development of full potential, talents and personality should be one of the aims of education (Article 15) [46]. Education (Additional Support for Learning) Act was passed in 2004, afterwards replaced by 2009 Act that established the need to identify and act on special needs of children and provide appropriate support (Paragraph 4) [47].

To this day, the most important legislation in the field of rights of autistic people passed in Scotland is the 10-year Scottish Strategy for Autism from 2011. Goal number 3 of the Strategy, ‘Whole-life journey’, highlights the need to identify and incorporate good-practice guidance in the field of education of autistic people [48]. This strategy also recognizes transition as an important period for autistic people, therefore recommendation number 18 deals with transition planning. Good practice guidance is to be established to help people with the condition make transitions in day-to-day life easier [48].

Policies passed by the UK Parliament that apply in Scotland along with policies introduced by Scottish Government form a combination that is consistent with Article 24 of CRPD on education. To policies that apply in the whole of the UK, Scotland’s addition in the form of a 10-year autism strategy is important as it shows that persons with the condition are noticed and their rights and needs are recognized by the Scottish Government.

#### 3.3.4 Northern Ireland

Similarly to other UK countries, in Northern Ireland, persons with disabilities should not be discriminated against when it comes to school admissions (Article 14 (1) [49]

The first autism-specific policy in Northern Ireland stated that after diagnosis, special support needs should be identified in numerous fields, including education (Paragraph 27)[50]. In 2011, the Parliament of Northern Ireland passed an act that required the government to create an autism strategy within 2 years [51]. The strategy was expected to show how needs of autistic people are addressed when it comes to education, health and social needs. In 2013, the autism strategy was created with 11 key themes and 16 priorities with education being one of them [52]. Northern Ireland is the first of UK- member countries to stress the importance of support given to parents along with the emphasis to get them involved. One of the priorities establishes that education should lead to improvement of preparation for life and work. The same priority mentions the importance of working together to improve early diagnosis of autism and identify and adopt good practice in the area of meeting the needs of people with the condition (Priority 8)[52]. According to the strategy progress report published in 2015, the Department for Employment and Learning offers services to autistic people, such as guidance to achieve highest level of education and advice on possible career paths (Paragraph 4.2) [52]. Moreover, a School Training Programme had been delivered to 3.500 persons with the condition (Paragraph 7.2) [52].

Although many pieces of UK legislation do not apply in Northern Ireland, they formulated acts, plans and strategies that are very autism-specific. Their first ASD Strategic Action Plan was the earliest autism-related strategy in the UK. Autism policies in Northern Ireland in the field of education are in full alignment with Article 24 of CRPD on education.

### 3.4 Poland

Starting in 1991, Act on Education System was passed by Polish Government that stated the right to education for everyone (Article 1(1) [53]. It is important to note that this Act was the first piece of legislation in the field of education after the democratic changes in 1989. It establishes that support should be given to children who need it and teaching methods should be tailored to pupils’ abilities (Article 1(4)[53]. Moreover, persons with disabilities have a right to attend either mainstream or special school, depending on their needs and preferences (Article 1(5) [53]. The Act also mentioned that free transport to school for children with disabilities should be organized, or parents should be refunded if transport is provided by them (Article 14a, Article 17(3a) [53]. Moreover, mainstream schools should be organized in a way that enables children with disabilities to be an active part of it (Article 17(1) [53]. In 1997, The Constitution of the Republic of Poland recalled the right to education for everyone (Article 70(1)) [54].

To this day there is no autism-specific legislation in Poland. However, in 2013, Charter for Persons with Autism was signed by Sejm (the lower house of the Polish parliament) which is based on the Charter for Persons with Autism adopted by the European Parliament in 1996 [55]. Autism-Poland sent the project to committees of the lower house of the Polish parliament such as Health Committee and the Committee on Justice and Human Rights. The parliament initially rejected the charter as ministries argued that autistic people fall under the disability laws and their rights are also included in many other documents (e.g. the CRPD). However, after discussions it was agreed that autistic people require specific provisions, and the Charter was adopted. Nevertheless, it is not binding, and its main aim is to bring attention to autism and highlight that autism specific needs should be taken into consideration in policy-making as well as when creating governmental programs and interventions. In regards to education, it is mentioned that education shall be easily accessible and free, tailored to specific needs of autistic children (Point 3) [55].

Policies in Poland are the most general of all countries analysed in this paper, as disability-specific and autism-specific policies in the field of education are absent. Basic rights, such as the right to education are provided, but there are no strategies in place to ensure this right is exercised. If there are no dedicated laws and specific legislation for autistic people, provisions are very general as not much can be drawn from the constitution itself. Such system makes it almost impossible for autistic people to claim the rights they ought to be entitled to.

### 3.5 France

Even before the UDHR, the Preamble to the French Constitution stated that there should be equal access to free, public education and vocational training provided by the state (Article 13)[56]. In 1975 a France Law was passed that introduced the legal concept of a person with disabilities, but no definition was established, what made the concept dependant on certain circumstances [57]. This law was then modified in 1996 and recognized that autism requires multidisciplinary support because of the varying needs of persons with the condition. According to this Law, the Government had a duty to submit a report including information on current care of autistic people as well as the prevalence of autism in children and adults (Article 3) [58].

In 2005, law on equal rights and opportunities, participation and citizenship of people with disabilities was passed by the French Parliament and was considered a landmark legislation [59]. Autism was not mentioned in this document, nevertheless it stated that needs, including education, of all people with disabilities need to be met as a form of compensation for the consequences of their disability (Article 11) [59]. Persons with disabilities should also be admitted to school closest to their home (Article 19) [59]. Teachers shall receive training on disability awareness and education of persons with disabilities (Article 19) [59].

To date, France has introduced three autism strategic plans. The 2005–2007 strategy aimed to increase school attendance of autistic children in mainstream settings [60]. Emphasis was also put on increasing teacher training, and a Autism Resource Centre was created to further increase support [60]. The 2008–2010 strategy also placed importance on improved teacher training in the field of autism [61]. The 2013–2017 strategy is currently in place and put more emphasis on education that two previous strategies [62]. The plan is divided into 5 strands: early detection, lifelong support, family support, research and training and raising awareness for professionals in the field of autism. Early detection of autism (at 18 months) combined with educational, behavioural and developmental support was mentioned in the plan (Strand 1)[62]. Action Sheet 33 builds on this goal and specifies that training should be provided to all professionals and should involve both types of staff: those that teach students in national education and those that fall under responsibility of local and regional authorities [62]. Strand 2 (B, 4) mentions support given to mainstream schooling by providing extra 550 places in special education and home care services (SESSADs) [62]. Interestingly, the EHESP French School of Public Health was also involved in setting up professional training to support a new vision on education and training of autistic people which in the past had been extensively informed through psychanalytical doctrine.

France introduced numerous policies that support autistic people in the field of education. High emphasis was put on teacher training what is also mentioned in Article 24 of CRPD on education. Similar to the UK and Spain, France created autism plans to ensure the rights of persons with the condition are fulfilled.

### 3.6 Spain

The Spanish Constitution from 1978 in Article 27(1) stated that everyone has a right to education and in Article 27(2) and that education should be directed at meeting their full potential [23]. Reaching potential of autistic people means their true abilities and passions, that may be hidden due to the condition, are discovered and nurtured so they can live their lives to the fullest. This is the first constitution to not only mention the right to education but also its aims. Moreover, Article 49 mentions the need to integrate people with disabilities as well as ensure special attention to their needs is given [23].

In 1982, Law on the Social Integration of the Handicapped was passed in Spain. Article 23(1) stated that people with disabilities should be integrated, attend mainstream schools and be provided with support (Law 13/1982). Article 23(2) stated that special education will only be provided if the child cannot be integrated into the mainstream school (Law 13/1982). Considering the wide spectrum of autism conditions, the effect of this legislation might be that autistic people might not receive the special treatment they require. Article 26(2) mentioned the aims of individualized education, such as; broadening knowledge and acquiring habits to enhance the chance of future independence and autonomy as well as developing personality and skills needed for future work so they are able to live a fulfilling life (Law 13/1982). Spain is the first country that recognized these rights early on, with the aforementioned articles in alignment with the CRPD but also directing focus and attention to the importance of self-development and fulfilment of people with disabilities as well as appropriate staff training. The Non-Discrimination Act (Law 51/2003) and the Equal Opportunities Act (Law 49/2007) are two other pieces of legislation that are not autism specific, nevertheless are relevant in the field of disability discrimination in Spain since they provided a strong basis against discrimination against autistic people.

In 2015 Spain adopted their first strategy for autism [63]. The strategy aims to improve the quality of life of autistic people and address their needs. Objective 2 of the strategy is focused on individualized and high quality education of autistic people whereas Objective 3 states that teaching methods should be adapted to be able to meet that goal of inclusion and personal development of autistic people [63]. Objective 4 mentions that students with autism should be encouraged to take part in educational as well as extra-curricular activities [63]. Objective 5 states that each autistic person will not only have different needs but also different aspirations and objectives, therefore the individualised education and evaluation should be provided [63]. The importance of identification, evaluation and implementation of good practice when it comes to education of autistic people is mentioned in Objective 6 [63]. Similarly to the UK, Spanish strategy in Objective 10 also recognizes difficulties that come with transitions between different stages of education, therefore continuous support should be provided and employment training should be increased [63]. Spanish disability policies have been developed and improved through the years are in full alignment with Article 24 of CRPD on education. Spain can be used as an example of policy-making that considers the rights and needs of autistic people. Nevertheless, more research should be conducted to establish whether the implementation and real effect of their strategies are successful and truly improves lives of persons with the condition.

## 4. Conclusion

There is a growing need to ensure the human rights of autistic people, such as that the right to inclusive education, are met. Autistic people should be given the opportunity to develop their full potential when given the right chance to flourish within a mainstream school setting. Individualized support in the field of education is required to enable them to lead life as autonomously and interdependently as possible.

The path dependency methodology used in this scoping review enabled us to explore interactions between policies from three different layers (International, EU, National) to answer our research question which was to establish current disability, SEN and education policies relevant for autistic people in four EU countries. The analysis of national policies in the UK, France, Poland and Spain showed they have been thoroughly influenced by international and EU policies through the years. Starting in 1948, the UDHR set standards that had to be met by all future national policies. This, partly, proved to be the case, as all countries analysed in this study had policies in place that guaranteed the right to education for everyone and stressed the importance of non-discrimination and equal opportunities for all. However, the scope and specificity of these documents differed between countries, with Poland having most general disability policy of all and being the only country without an autism strategy in place, whereas the UK, France and Spain had autism-specific plans present that were in alignment with Article 24 of CRPD. All UK countries had disability and autism-specific laws and legislation in place, nevertheless the approach and plan of action differed between its member countries. Our research showed that countries have increasingly noticed the importance and uniqueness of autistic people and progressively adapt their laws and strategies to suit their needs. Regardless, educational opportunities for autistic people remain a challenge across the EU. Although policies that ensure the provision of adequate services for autistic people exist, the availability and access to such services are questionable with wide variations in quality and access across the EU.

This study provided vital information on the right to education of autistic people in the UK, France, Poland and Spain. The scope of this study only included four countries, therefore the results cannot be generalized and clear conclusion on the average level of the fulfilment of the right to education cannot be drawn. More countries should be analysed to get a better picture of the situation across the EU. Additionally, since this is the first in a series of studies that map SEN policy in the EU, the findings have not been able to be triangulated to ensure reliability. Furthermore, the initial pool of identified studies has not been examined by other authors, meaning the reliability of the screening process cannot be guaranteed. More research should also be conducted to establish whether strategies that are in place have an effect on autistic children, such as improved learning, skills and higher rates of participation in education. To the best of our knowledge, there are no previous studies that have examined whether education of autistic people in EU countries is directed to development of their talents, creativity and provides them with skills they need to successfully progress into employment. To this day, the research in the field of education and autism policies in the EU as well as globally is scarce and remains an important gap in autism research. It is for this reason that this study aimed to review existing information as well as attract interest to conduct more research in this field in the future.

## Supporting information

S1 File Included studies. (PDF)

S2 File PRISMA autism policy checklist. (DOC)
