# Supplementary material for: Autism and the right to education in the EU: Policy mapping and scoping review of the United Kingdom, France, Poland and Spain
Source: PLoS One. 2018 Aug 30;13(8):e0202336. doi: 10.1371/journal.pone.0202336 (PMC6116926; doi:10.1371/journal.pone.0202336)
Supplement: S1 File — (PDF) [file pone.0202336.s001.pdf]

1 10 year Scottish Strategy for Autism (2011). Available at: http://www.gov.scot/Resource/Doc/361926/0122373.pdf.

2 Act on Education System (1991). Available at: http://dziennikustaw.gov.pl/du/1991/s/95/425.

3 ASD Strategic Action Plan (2009). Available at: http://www.hscboard.hscni.net/download/PUBLICATIONS/AUTISTIC SPECTRUM DISORDER/DHSSPS ASD Strategic Action Plan June 2009 - PDF 373KB.pdf.

4 Autism Act (2009). Available at: http://www.legislation.gov.uk/ukpga/2009/15.

5 'Autism Act (Northern Ireland)' (2011). Available at: http://www.legislation.gov.uk/nia/2011/27/pdfs/nia_20110027_en.pdf.

6 Charter for Persons with Autism (1992). Available at: http://www.autismeurope.org/files/files/charter-for-persons-with-autism.pdf.

7 Charter for Persons with Autism Poland (2013). Available at: http://orka.sejm.gov.pl/opinie7.nsf/nazwa/380_u/$file/380_u.pdf.

8 Children and Families Act (2014). Available at: http://www.legislation.gov.uk/ukpga/2014/6/contents/enacted.

9 Constitution of The Republic of Poland (1997). Available at: http://www.sejm.gov.pl/prawo/konst/angielski/kon1.htm.

10 CRPD (2008). Available at: http://www.ohchr.org/EN/HRBodies/CRPD/Pages/ConventionRightsPersonsWithDisabilities.aspx.

11 Declaration of the Rights of the Child (1959). doi: 10.1017/S0020860400013243.

12 Declaration on the Rights of Disabled Persons (1975). Available at: http://www.ohchr.org/Documents/ProfessionalInterest/res3447.pdf.

13 Declaration on the Rights of Mentally Retarded Persons (1971). Available at: http://www.ncbi.nlm.nih.gov/pubmed/10239923.

14 Education (Additional Support for Learning) (Scotland) Act 2004 (2004). Available at: http://www.legislation.gov.uk/asp/2004/4/pdfs/asp_20040004_en.pdf.

15 Equality Act (2010). doi: ISBN 978-0-10-541510-7.

16 European Disability Strategy 2010-2020 (2010). doi: 10.1017/CBO9781107415324.004.

17 Fulfilling and Rewarding Lives (2010). Available at: http://webarchive.nationalarchives.gov.uk/20130107105354/http://www.dh.gov.uk/prod_consum_dh/groups/dh_digitalassets/@dh/@en/@ps/documents/digitalasset/dh_113405.pdf.

18 Human Rights Act (1998). Available at: http://www.legislation.gov.uk/ukpga/1998/42.

19 Law no. 2005-102 (2005).

20 Law no. 75-535 (1975).

21 Law no. 96-1076 (1996).

22 Preamble to the Constitution of 27 October (1946). Available at: http://www.conseil-constitutionnel.fr/conseil-constitutionnel/root/bank_mm/anglais/cst3.pdf.

23 Refreshed Autistic Spectrum Disorder (ASD) Strategic Action Plan created for 2016-2020 (2016). Available at: http://gov.wales/docs/dhss/publications/161205asd-delivery-en.pdf.

24 Spanish Constitution (1978). Available at: http://www.congreso.es/portal/page/portal/Congreso/Congreso/Hist_Normas/Norm/const_espa_texto_ingles_0.pdf.

25 Spanish Strategy For ASD (2015). Available at: https://www.msssi.gob.es/ssi/discapacidad/informacion/estrategiaEspanolaAutismo.htm.

26 Special Educational Needs and Disability (Northern Ireland) Order (2005).

27 Special Educational Needs and Disability Act (2001). Available at: http://www.legislation.gov.uk/ukpga/2001/10/contents.

28 Standards in Scotland's Schools Act (2000). Available at: http://www.legislation.gov.uk/asp/2000/6.

29 Statutory Framework for the Early Years Foundation Stage (2014). doi: 00266-2008BKT-EN.

30 Statutory Guidance - Special Educational Needs and Disability Code of Practice: 0 to 25 years (2015). doi: DFE-00205-2013.

31 Statutory Guidance Fulfilling and Rewarding Lives (2010). Available at: https://www.gov.uk/government/publications/implementing-fulfilling-and-rewarding-lives.

32 The Autism Strategy (2013 – 2020) Action Plan (2013 – 2016) (2013). Available at: https://www.health-ni.gov.uk/sites/default/files/publications/dhssps/autism-strategy-progress-report-2015.pdf.

33 The Autistic Spectrum Disorder (ASD) Strategic Action Plan for Wales (2009). Available at: http://www.wales.nhs.uk/documents/ASD-strategy.pdf.

34 The Charter of Fundamental Rights of the European Union (2000). doi: 10.1108/03090550310770974.

35 The First Autism Plan (2005). Available at: http://www.psydoc-france.fr/Professi/Autisme/Plans/PlanAutisme2005-2006.pdf.

36 The Second Autism Plan (2008). Available at: http://www.cnsa.fr/documentation/plan_autisme_2008.pdf.

37 The Third Autism Plan (2013). Available at: http://www.cnsa.fr/documentation/plan-autisme2013.pdf.

38 The Treaty on the Functioning of the European Union (2007). Available at: http://eur-lex.europa.eu/legal-content/EN/TXT/?uri=celex%3A12012E%2FTXT.

39 Think Autism (2014). Available at: http://www.autism.org.uk/~/media/NAS/Documents/News-and-events/News-from-the-NAS/Think Autism Strategy.ashx.

40 Together for Mental Health. A Strategy for Mental Health and Wellbeing in Wales (2012). Available at: http://gov.wales/topics/health/nhswales/plans/mental-health/?lang=en.

41 Treaty on the European Union (2012). Available at: http://eur-lex.europa.eu/legal-content/EN/TXT/?uri=celex%3A12012M%2FTXT.

42 UDHR (1948). Available at: http://www.ohchr.org/EN/UDHR/Documents/UDHR_Translations/eng.pdf.

43 WHO (2011) World Report on Disability. Available at: http://www.who.int/disabilities/world_report/2011/report.pdf.

44 WHO (2016) Demographic and Socioeconomic Statistics. Available at: http://apps.who.int/gho/data/view.main.POP2040?lang=en.

45 Written Declaration on Autism (2015). Available at: http://www.europarl.europa.eu/sides/getDoc.do?pubRef=-//EP//NONSGML+WDECL+P8-DCL-2015-0030+0+DOC+PDF+V0//EN&language=EN.
