# Supplementary material for: Autism and the right to education in the EU: Policy mapping and scoping review of the United Kingdom, France, Poland and Spain
Source: PLoS One. 2018 Aug 30;13(8):e0202336. doi: 10.1371/journal.pone.0202336 (PMC6116926; doi:10.1371/journal.pone.0202336)
Supplement: S2 File — (DOC) [file pone.0202336.s002.doc]

| **Section/topic** | **#** | **Checklist item** | **Reported on page #** |
| --- | --- | --- | --- |
| **TITLE** | | |  |
| Title | 1 | Identify the report as a systematic review, meta-analysis, or both. | **Page 1:** “Autism and the Right to Education in the EU: Policy Mapping and Scoping Review of the United Kingdom, France, Poland and Spain (page1).”  This is a scoping review and we have included this in the title. |
| **ABSTRACT** | | |  |
| Structured summary | 2 | Provide a structured summary including, as applicable: background; objectives; data sources; study eligibility criteria, participants, and interventions; study appraisal and synthesis methods; results; limitations; conclusions and implications of key findings; systematic review registration number. | **Page 2:** Introduction: People diagnosed with autism have different educational needs that should be met to allow them to develop their full potential. Education and disability policies remain within competencies of EU Member States, with current educational standards and provisions for people with autism not widely understood. This scoping review aims to map EU and national policies with the goal of scoping the level of fulfilment of right to education of persons with autism.  Methods: Four EU countries (United Kingdom, France, Poland and Spain) were included in this scoping review study. Governmental policies in the field of education, SEN and disability law were included. Path dependency framework was used for data analysis; a net of inter-dependencies between international, EU and national policies was created.  Results and discussion: Each country created policies where the right to free education without discrimination is provided. Poland does not have an autism strategy, whereas the UK, France and Spain have policies specifically designed for persons with the condition. All UK countries created different autism plans, nevertheless all aim to reach the same goal - inclusive education for children with the condition that leads to the development of their full potential.  Conclusion: Policy-making across Europe in the field of education has been changing through the years in favour of persons with autism. Today their rights are noticed and considered, but there is still room for improvement. Results showed that approaches and policies vastly differ between countries, more Member States should be analysed in a similar manner to gain a broader and clearer view with a special focus on disability rights in Central and Eastern Europe. |
| **INTRODUCTION** | | |  |
| Rationale | 3 | Describe the rationale for the review in the context of what is already known. | **Page 4-5:** “Although several areas of law are harmonized in the EU, public health remains within the competences of the Member States (Article 168)(15). In this paper we hypothesize that the lack of harmonization in public health and education policy and common standard of policy making in EU countries has resulted in a gap of delivery of SEN provision and the fulfilment of the right to education of autistic people across Europe. In order to evaluate this we included four European countries, namely the United Kingdom, France, Poland and Spain, countries as part of a wider project to map all 28 EU Member States SEN and ASC policy; aiming to create a comparative policy analysis that will culminate in a report of the differences that might be observed in ASC policy across Europe. The work carried out in this scoping review aims to first map International, EU and national ASC policies in the field of education across these four countries, to then expand all 28 EU Member States as part of a larger project. We use a policy path dependency methodology that links both the precedents of the UDHR and CRPD as main theoretical frameworks of reference.” |
| Objectives | 4 | Provide an explicit statement of questions being addressed with reference to participants, interventions, comparisons, outcomes, and study design (PICOS). | **Page 4-5:** “We aim to show main policy outcomes and the levels of interdependency between the different layers of policy developments to gain a clear view on the level of fulfilment of right to education and public health impact of autistic people across four EU countries.” |
| **METHODS** | | |  |
| Protocol and registration | 5 | Indicate if a review protocol exists, if and where it can be accessed (e.g., Web address), and, if available, provide registration information including registration number. | N/A. There is no protocol since this is a scoping review. |
| Eligibility criteria | 6 | Specify study characteristics (e.g., PICOS, length of follow-up) and report characteristics (e.g., years considered, language, publication status) used as criteria for eligibility, giving rationale. | **Page 6:** “We included the United Kingdom (64.72 million people) (divided between England, Wales, Scotland and Northern Ireland), France (64.40 million people), Poland (38.61 million people) and Spain (46.12 million people) (20) as a sample of different policy initiatives across the EU and also considering that these countries represents a large percentage (42.36%) of the total EU population and this would allow us to comparatively asses a large proportion of people that might be affected in the EU by ASC and SEN policy. We included these four countries as the initial step to map all 28 EU Member States ASC policy, work that will be carried out in parallel and will result in a EU wide report. The scope of ASC policies was narrowed to only include policies that relate to the national education system, the right to education, special needs education, and disability laws. Additionally, policies and documents relating to autism and educational policy of those younger than 18 years, with any comorbid health condition in any setting were eligible for inclusion. Legal documents provided by governments were included, whereas programs and strategies developed by non-governmental organizations were excluded. Furthermore, legislation was eligible for inclusion as long as it was published after 1945. Constitutions were included regardless of publication date, because of their fundamental role in legislation.” |
| Information sources | 7 | Describe all information sources (e.g., databases with dates of coverage, contact with study authors to identify additional studies) in the search and date last searched. | **Page 7:** “There was just one governmental source for the United Nations (http://www.ohchr.org), two for the EU ([http://eur-lex.europa.eu](http://eur-lex.europa.eu/); http://www.europarl.europa.eu), five for the UK and its constituent countries ([http://www.legislation.gov.uk](http://www.legislation.gov.uk/); [http://www.gov.scot](http://www.gov.scot/); [http://gov.wales](http://gov.wales/); [http://www.wales.nhs.uk](http://www.wales.nhs.uk/); https://www.health-ni.gov.uk), three for Poland ([http://dziennikustaw.gov.pl](http://dziennikustaw.gov.pl/); [http://orka.sejm.gov.pl](http://orka.sejm.gov.pl/); http://www.sejm.gov.pl), two for France ([http://www.conseil-constitutionnel.fr](http://www.conseil-constitutionnel.fr/); http://www.cnsa.fr) and two for Spain ([http://www.congreso.es](http://www.congreso.es/);<https://www.msssi.gob.es/>). Moreover, no time limit was used during the searches, as the goal was to create a timeline of policies. The second step was to develop a multi-faceted search strategy to search through electronic databases (PubMed, Google Scholar) using a combination of text words and index terms.” |
| Search | 8 | Present full electronic search strategy for at least one database, including any limits used, such that it could be repeated. | **Page 7:** “The first step or identification stage was to extract relevant data from governmental policies and legislation that address the right to education of autistic people. Data was taken directly from their original sources in legal documents from the four countries included in this scoping review. No limits were put on language and foreign documents that were not translated into English. There was just one governmental source for the United Nations (http://www.ohchr.org), two for the EU ([http://eur-lex.europa.eu](http://eur-lex.europa.eu/); http://www.europarl.europa.eu), five for the UK and its constituent countries ([http://www.legislation.gov.uk](http://www.legislation.gov.uk/); [http://www.gov.scot](http://www.gov.scot/); [http://gov.wales](http://gov.wales/); [http://www.wales.nhs.uk](http://www.wales.nhs.uk/); https://www.health-ni.gov.uk), three for Poland ([http://dziennikustaw.gov.pl](http://dziennikustaw.gov.pl/); [http://orka.sejm.gov.pl](http://orka.sejm.gov.pl/); http://www.sejm.gov.pl), two for France ([http://www.conseil-constitutionnel.fr](http://www.conseil-constitutionnel.fr/); http://www.cnsa.fr) and two for Spain ([http://www.congreso.es](http://www.congreso.es/);<https://www.msssi.gob.es/>). Moreover, no time limit was used during the searches, as the goal was to create a timeline of policies.”  “The second step was to develop a multi-faceted search strategy to search through electronic databases (PubMed, Google Scholar) using a combination of text words and index terms. Words included in our search were “autism”; “disability”; “SEN”; “education”; “law”; “policy” and the following combinations “autism & law”; “autism & policy”; “autism & SEN”; “autism & education”; “autism & disability”; “SEN & policy”; “SEN & law”; “disability & law”; “disability & policy”.”  “We adapted the search strategy for each database, and then in a third step of screening, we amalgamated both policy and academic publications. Legal and policy documents provided by the governments were included after testing in the eligibility stage, whereas programs and strategies developed by non-governmental organizations were excluded.”  “The fourth step of our search strategy was to obtain further information through searching reference lists and grey literature. Policy documents and governmental strategies in selected countries were delineated to the EU disability and educational policy. In case mentioned documents were not present, general disability policies and legislation were analysed. This was built on an appraisal of three searches: one looking for ASC and educational policy in the international level, one for the EU and one looking for the individual countries.”  “The fifth step was merging these three geographical searches into a single data repository for the purpose of our scoping review.” |
| Study selection | 9 | State the process for selecting studies (i.e., screening, eligibility, included in systematic review, and, if applicable, included in the meta-analysis). | **Page 7:** “We included these four countries as the initial step to map all 28 EU Member States ASC policy, work that will be carried out in parallel and will result in a EU wide report. The scope of ASC policies was narrowed to only include policies that relate to the national education system, the right to education, special needs education, and disability laws. Additionally, policies and documents relating to autism and educational policy of those younger than 18 years, with any comorbid health condition in any setting were eligible for inclusion. Legal documents provided by governments were included, whereas programs and strategies developed by non-governmental organizations were excluded. Furthermore, legislation was eligible for inclusion as long as it was published after 1945. Constitutions were included regardless of publication date, because of their fundamental role in legislation.” |
| Data collection process | 10 | Describe method of data extraction from reports (e.g., piloted forms, independently, in duplicate) and any processes for obtaining and confirming data from investigators. | **Page 7:** “This was built on an independent appraisal of three searches: one looking for ASC and educational policy in the international level one for the EU and one looking for the individual countries. These three searches were merged into a single strategy and adapted for the purpose of our scoping review. All data collection and analysis was based at the Autism Research Centre and Institute of Public Health University of Cambridge.” |
| Data items | 11 | List and define all variables for which data were sought (e.g., PICOS, funding sources) and any assumptions and simplifications made. | We performed a PICOS assessment for the variables sought.   - Patient, Population or Problem   We defined the characteristics of the patient or population as those autistic people in education that might or not require special education needs in Spain, France, United Kingdom and Poland.   - Intervention   The intervention that we included in this work is education and more specifically special education needs.   - Comparison   We don’t use the a direct comparisons, but we use a human rights approach in which we discuss up to what extent special educational needs have been included in policy making and more specifically in autism policy.   - Outcome   Our main outcome measure is a policy mapping of autism policy at the international, European and national level in Spain, France, United Kingdom and Poland. |
| Risk of bias in individual studies | 12 | Describe methods used for assessing risk of bias of individual studies (including specification of whether this was done at the study or outcome level), and how this information is to be used in any data synthesis. | N/A. There is no bias analysis since this is a scoping review. We do describe the short falls of the paper though. |
| Summary measures | 13 | State the principal summary measures (e.g., risk ratio, difference in means). | N/A |
| Synthesis of results | 14 | Describe the methods of handling data and combining results of studies, if done, including measures of consistency (e.g., I^2^) for each meta-analysis. | N/A. No meta analysis was conducted |

Page 1 of 2

| **Section/topic** | **#** | **Checklist item** | **Reported on page #** |
| --- | --- | --- | --- |
| Risk of bias across studies | 15 | Specify any assessment of risk of bias that may affect the cumulative evidence (e.g., publication bias, selective reporting within studies). | Page 20. “The scope of this study only included four countries, therefore the results cannot be generalized and clear conclusion on the average level of the fulfilment of the right to education cannot be drawn. More countries should be analysed to get a better picture of the situation across the EU. More research should also be conducted to establish whether strategies that are in place have an effect on children with autism, such as improved learning, skills and higher rates of participation in education.” |
| Additional analyses | 16 | Describe methods of additional analyses (e.g., sensitivity or subgroup analyses, meta-regression), if done, indicating which were pre-specified. | N/A |
| **RESULTS** | | |  |
| Study selection | 17 | Give numbers of studies screened, assessed for eligibility, and included in the review, with reasons for exclusions at each stage, ideally with a flow diagram. | **Page 8:** “We identified 18 sources through database searches (PubMed and Google Scholar) and 91 additional records through governmental sources at the study identification stage. At this stage we observed no duplicates and therefore we included 109 studies and policy documents for the screening stage. During the screening of 103 records (6 records were excluded since they were different in scope), we excluded 54 records that did not meet our inclusion criteria after which we were able to include 49 records assessed for eligibility with four articles and or policy documents excluded at this stage. The final number of studies included in our qualitative analysis was 45.”  **Page 9:** International level, **Page 10:** EU level, **Page 12:** UK and England, **Page 15:** Wales, **Page 16:** Scotland, **Page17:** Northern Ireland, **Page 18:** Poland, **Page 20:** France, **Page 21:** Spain. |
| Study characteristics | 18 | For each study, present characteristics for which data were extracted (e.g., study size, PICOS, follow-up period) and provide the citations. | **Pages 6 – 19:** This was a scoping review therefore no systematic review was performed. We have provided a list of all data and links for all documents included. Please refer to this for specific details. |
| Risk of bias within studies | 19 | Present data on risk of bias of each study and, if available, any outcome level assessment (see item 12). | N/A The paper only used legal and policy documents therefore no outcome level assessment was performed. |
| Results of individual studies | 20 | For all outcomes considered (benefits or harms), present, for each study: (a) simple summary data for each intervention group (b) effect estimates and confidence intervals, ideally with a forest plot. | N/A The paper only used legal and policy documents therefore no outcome level assessment was performed. |
| Synthesis of results | 21 | Present results of each meta-analysis done, including confidence intervals and measures of consistency. | N/A The paper only used legal and policy documents therefore no outcome level assessment was performed. |
| Risk of bias across studies | 22 | Present results of any assessment of risk of bias across studies (see Item 15). | N/A The paper only used legal and policy documents therefore no outcome level assessment was performed. |
| Additional analysis | 23 | Give results of additional analyses, if done (e.g., sensitivity or subgroup analyses, meta-regression [see Item 16]). | N/A The paper only used legal and policy documents therefore no outcome level assessment was performed. |
| **DISCUSSION** | | |  |
| Summary of evidence | 24 | Summarize the main findings including the strength of evidence for each main outcome; consider their relevance to key groups (e.g., healthcare providers, users, and policy makers). | **Page 23-24:** “The analysis of national policies in the UK, France, Poland and Spain showed they have been thoroughly influenced by international and EU policies through the years. Starting in 1948, Universal Declaration of Human Rights set standards that had to be met by all future national policies. This, partly, proved to be the case, as all countries analysed in this study had policies in place that guaranteed the right to education for everyone and stressed the importance of non-discrimination and equal opportunities for all. However, the scope and specificity of these documents differed between countries, with Poland having most general disability policy of all and being the only country without an autism strategy in place, whereas the UK, France and Spain had autism-specific plans present that were in alignment with Article 24 of CRPD.” |
| Limitations | 25 | Discuss limitations at study and outcome level (e.g., risk of bias), and at review-level (e.g., incomplete retrieval of identified research, reporting bias). | **Page 24:** “The scope of this study only included four countries, therefore the results cannot be generalized and clear conclusion on the average level of the fulfilment of the right to education cannot be drawn. More countries should be analysed to get a better picture of the situation across the EU. More research should also be conducted to establish whether strategies that are in place have an effect on children with autism, such as improved learning, skills and higher rates of participation in education.” |
| Conclusions | 26 | Provide a general interpretation of the results in the context of other evidence, and implications for future research. | **Page 24:** “All UK countries had disability and autism-specific laws and legislation in place, nevertheless the approach and plan of action differed between its member countries. Our research showed that countries have increasingly noticed the importance and uniqueness of persons with autism and progressively adapt their laws and strategies to suit their needs. Nevertheless, educational opportunities for people with the condition are still insufficient and remain a challenge across the EU. Although policies that ensure the provision of adequate services for persons with ASC exist, the availability and access to such services are questionable with wide variations in quality and access across the EU.” |
| **FUNDING** | | |  |
| Funding | 27 | Describe sources of funding for the systematic review and other support (e.g., supply of data); role of funders for the systematic review. | **Page 1:** “Dr Andres Roman-Urrestarazu’s work received funding from the Gillings Fellowship in Global Public Health and Autism Research, Grant Award YOG054. Dr Rosemary Holt and Dr Amber Ruigrok received funding from Innovative Medicines Initiative 2 Joint  Undertaking (JU) under grant agreement No 777394. The JU receives support from the European Union’s Horizon 2020 research and innovation programme and EFPIA and AUTISM SPEAKS, Autistica, SFARI.” |

*From:*  Moher D, Liberati A, Tetzlaff J, Altman DG, The PRISMA Group (2009). Preferred Reporting Items for Systematic Reviews and Meta-Analyses: The PRISMA Statement. PLoS Med 6(7): e1000097. doi:10.1371/journal.pmed1000097

For more information, visit: **www.prisma-statement.org**.

Page 2 of 2
